# Application of a sEMG hand motion recognition method based on variational mode decomposition and ReliefF algorithm in rehabilitation medicine

**DOI:** 10.1371/journal.pone.0314611

**Published:** 2024-11-27

**Authors:** Yue Yuan

**Affiliations:** School of Information Engineering, Shenyang University, Shenyang, Liaoning, China; Universita Politecnica delle Marche Facolta di Ingegneria, ITALY

## Abstract

Hand motion intention recognition has been considered as one of the crucial research fields for prosthetic control and rehabilitation medicine. In recent years, surface electromyogram (sEMG) signals that directly reflect human motion information are ideal input sources for prosthetic control and rehabilitation. However, how to effectively extract components from sEMG signals containing abundant limb movement information to improve the accuracy of hand recognition still is a difficult problem. To achieve this goal, this paper proposes a novel hand motion recognition method based on variational mode decomposition (VMD) and ReliefF. First, VMD is used to decompose the sEMG signal into multiple variational mode functions (VMFs). To efficiently extract the intrinsic components of the sEMG, the recognition performance of different numbers of VMFs is evaluated. Then, four features representing hand motion intentions are extracted from the VMFs to form the initial feature space. Next, the ReliefF algorithm is used to remove redundant features from the feature space. In order to select a feature space that can effectively reflect the intention of hand movements, the hand movement recognition performance of 8 low-dimensional feature spaces is evaluated. Finally, three machine learning methods are used to recognize hand movements. The proposed method was tested on the sEMG for Basic Hand movements Data Set and achieved an average accuracy of 99.14%. Compared with existing research, the proposed method achieves better hand motion recognition performance, indicating the potential for healthcare and rehabilitation applications.

## 1. Introduction

The number of people disabled by hand amputations has increased significantly around the world in recent years [1]. Over the past decade, 209 elderly patients underwent major limb amputations, with 48% of these cases attributed to diabetes and the remaining cases resulting from accidents and other diseases [2]. Disability has been identified as a significant issue impacting individuals’ lives within the Australia Indonesia Partnership for Economic Governance [2]. Given the substantial and growing population of individuals with hand disabilities, finding solutions to these challenges is of utmost importance [3]. One potential solution to this problem is to develop a prosthetic hand that mimics the functions of a natural hand. Prosthetic hand can replace lost limbs and help disabled people regain some level of physical function [4]. Prosthetic hand can provide basic grasping and other functions, enabling people with disabilities to perform daily activities and improve their quality of life. Through the use of prosthetic hand, people with disabilities can increase their independence, take care of themselves, carry out daily activities, participate in social and professional activities, etc., so that they can better adapt to the social environment and improve their ability to take care of themselves [5]. Prosthetic hand provides a way to restore bodily integrity and function, helping to reduce the psychological stress and emotional distress of people with disabilities. By restoring some physical function, people with disabilities can better adjust to the disabled state and have an enhanced sense of self-esteem and self-worth [6]. Prosthetic hand enabled people with disabilities to communicate and interact with others more confidently, increasing the possibility of social interaction [7]. People with disabilities can use prosthetic hand to participate in various physical and athletic activities which helps to improve physical health and mental state, as well as enhance physical fitness [8]. Although the prosthetic hand has so many advantages for the disabled, there are still many key problems in the practical application of the prosthetic hand for the disabled. Before creating a prosthetic hand, it is crucial to determine an appropriate input signal for controlling its movements. Several studies have explored controlling hand movements using computer vision and wearable sensors [9–11].

Various methods are available for analyzing images captured by optical devices in the context of hand movement recognition [12–15]. Reference [10] proposed a deep learning model specifically for sign language recognition using gestures captured by RGB cameras. Reference [16] introduced a gesture recognition system that combines RGB images and depth information while employing a feature extraction method based on quaternion algebra. This approach utilizes quaternion algebra for holistic processing, considering four components for gesture recognition. Reference [17] used RGB-D data for sign language recognition, and proposed four-stream CNN uses 4 inputs in the training space, in the testing space, only RGB and RGB temporal data are used for prediction based on the trained model. However, hand motion recognition using optical devices is often influenced by various factors, such as camera angle, occlusion and illumination intensity [18,19].

Wearable sensors that directly capture signals generated by hand motions are gaining significant attention for hand motion recognition [20–23] when compared to optical devices. Notably, wearable sensors commonly include sEMG signal sensors, inertial measurement units (IMU), and gyroscopes, as demonstrated in previous research [24]. Reference [25] utilized multiple IMU sensors for gesture recognition. Their approach involved frequency convolutional neural networks and temporal neural networks to extract representative features from IMU signals within a sliding window, facilitating the recognition of various gesture types. The proposed method was evaluated on basketball official referee signals (ORSs), encompassing 65 gestures, including both large and small motion gestures. Reference [26] proposes a multimodal framework combining IMU and microphone unit to recognize fourteen gestures. This method extracts 7873 features from each sensor, and uses minimum redundancy maximum relevance to select valuable features and feed them into the classifier to recognize hand movements. Reference [27] proposes a restricted column energy neural network capable of processing time-correlated data from IMU sensors to recognize gestures. The method is verified on the FPGA platform with a recognition accuracy of 98.6%. Reference [28] proposed a training system that combines electromyography, force myography, and inertial sensing to estimate natural movements of the upper body. Validation of the training system was conducted on datasets from three stroke patients and eight healthy subjects, demonstrating its ability to improve ADL performance. Compared to signals obtained from other wearable sensors, sEMG signals directly reflect the electrophysiological responses of limb movements, offering inherent advantages in predicting limb motion [29–35]. Reference [36] developed an EMG-based hand/finger gesture classifier using a machine learning approach and fixed electrode placement. The method was evaluated on a dataset from ten healthy subjects, and the experimental results demonstrated that artificial neural networks achieved an average accuracy of 0.940, followed by support vector machines (0.876), random forest (0.831), and logistic regression (0.539). Reference [37] proposed a method for feature extraction of sEMG signals based on muscle region activation. They combined the newly extracted features with sample entropy features and wavelength features. The fused features were then used in conjunction with the K-nearest neighbors (KNN) to recognize and classify hand movement patterns among multi-object groups. It can be observed that the sEMG signal has emerged as a primary input signal in the field of hand motion recognition.

The sEMG signal is a bioelectric signal generated by the simultaneous contraction and relaxation of multiple muscles, which directly reflects the body’s physical activity [38,39]. The sEMG signals contain the activity information of different muscles, which often overlapping, so it is a major challenge to separate the independent activity components of each muscle to extract the subcomponents generated by a specific muscle or muscle fiber group. The separation of subcomponents is crucial for subsequent feature extraction and pattern recognition, and helps to improve the accuracy of classifiers in hand movements recognition. Although many studies have focused on using sEMG signals for hand movements recognition, there are still few studies specifically focusing on the basic components of sEMG signals in the context of hand movements recognition [40–42]. How to accurately extract the necessary components of sEMG signals is also an ongoing research area [43–45]. Reference [46] introduces a method to extract useful information from forearm surface EMG using Stockwell transform to improve the accuracy of hand motion recognition. The proposed method involves applying S-transform to obtain eigenvectors from the forearm sEMG signals. Reference [47] compared the processing capabilities of continuous-time wavelet transform, short-time Fourier transform, and scale-averaged wavelet transform for sEMG signals. Their test results demonstrate that the convolutional recurrent neural network utilizing the scale-averaged wavelet transform and overlapping window outperforms the other methods in both real-time and stationary tests. Reference [48] presented a sEMG signal processing approach based on wavelet transform and wavelet weighted permutation entropy (WWPE). The method involves decomposing and preprocessing the collected upper limb-related sEMG signals using wavelet transform to obtain subcomponent. Weighted permutation entropy is extracted from the wavelet sub-bands, constructing a feature set for classification. A support vector machine classifier is then employed, utilizing the WWPE feature set to recognize seven hand movements. Reference [49] proposed tunable-Q wavelet transform for feature extraction from surface EMG signals. The resulting feature set was used as input to the classifier for hand movement identification. Reference [50] features extracted from intrinsic mode functions obtained by decomposing surface EMG signals using empirical mode decomposition are fed into a simple linear classifier for hand motion classification. Among the above methods, the performance of the decomposition method based on wavelet transform is affected by factors such as correlation coefficient and wavelet basis function, resulting in the inability of the decomposed sEMG signal sub-components to effectively characterize hand movement [51]. In addition, the empirical mode decomposition (EMD) method is prone to modal aliasing when processing sEMG signals, thus affecting the decomposition effect [50]. Related studies have shown that compared with the EMD method, the ensemble empirical mode decomposition (EEMD) can improve the signal-to-noise ratio and consistency of the sub-components and effectively suppress the modal aliasing phenomenon by introducing Gaussian white noise into the original sEMG signal and performing multiple averaging processes. However, the EEMD method has a large amount of calculation, and the modal components are not easy to control, which may cause the function to not converge, thereby affecting the decomposition accuracy of the sEMG signal. In contrast, the variational mode decomposition (VMD) has better robustness to noise by finding the optimal solution of the constrained variational model, and its decomposition results are more stable. In addition, the VMD can decompose the sEMG signal into narrow bandwidth sub-components, thereby avoiding modal aliasing and extracting the essential components more effectively [52]. Reference [52] utilized VMD to decompose the sEMG signal into multiple variate mode functions (VMFs) and calculated the composite permutation entropy index form each VMFs as a feature. Experimental results show that the average accuracy of hand movements combined with Laplacian score and Bagging classifier is 94.28%. Therefore, inspired by the performance comparison of various sEMG signal decomposition methods, this work adopts the VMD method to extract the basic components of sEMG signals, thereby improving the performance of hand movement recognition.

Another essential aspect of hand movement recognition is the extraction of effective features that represent hand movements [52]. Prior studies [53–57] have demonstrated that scholars typically consider time-domain (TD) and frequency-domain (FD) features for processing sEMG signals. TD features commonly include mean square error (VAR) and mean absolute value (MAV). Common FD features encompass median frequency (MDF), mean power (MNP), and mean frequency (MNF), among others [58,59]. In a study conducted by [59] various TD and FD features were extracted from sEMG signals to assess their effectiveness in decoding hand movements. However, these features alone were insufficient for accurately characterizing sEMG signals. One approach to address this limitation is to consider entropy as a measure of signal complexity. Previous studies [60,61] has explored the extraction of entropy features from sEMG signals to represent them effectively and reliably. Permutation entropy (PeEn), which quantifies the complexity of a time series, has been found to possess fast calculation speed, strong anti-interference ability, and good robustness, making it suitable for nonlinear signals [61]. Thus, incorporating permutation entropy features into hand motion recognition based on sEMG signals holds potential for improved performance.

According to the aforementioned analysis, sEMG signals contain rich information about hand movements. To improve the accuracy of hand movement recognition, it is necessary to decompose sEMG signals and extract subcomponents of different frequencies. At the same time, since there may be a lot of irrelevant information in the extracted features, it is also crucial to filter out the features most relevant to hand movement recognition to reduce the computational complexity. The objective of this study is to effectively extract informative components from sEMG signals, which contain rich limb movement information, in order to enhance the accuracy of hand movement recognition. The proposed approach involves employing VMD for signal decomposition into multiple VMFs. From these VMFs, features such as MAV, MDF, MNF, and PeEn are extracted. Redundant features are eliminated using the ReliefF algorithm, and machine learning techniques are employed for precise hand movement recognition. This method has fast response capabilities while ensuring accuracy. Comparisons with existing research highlight the significant implications of this proposed method for the advancement of human-computer interaction.

The contributions of this work are as follows: A novel method combining VMD with ReliefF is proposed, which provides innovative ideas for sEMG signal processing and hand motion recognition. The hand motion recognition performance of VMD with different numbers of VMFs is evaluated. The recognition performance of the proposed method in different low-dimensional feature spaces selected by the ReliefF algorithm is compared. The method of combining VMD and ReliefF proposed in this work achieves the accuracy of 99.14%, which is significantly better than the existing research.

In this work, the decomposition characteristics of VMD effectively reduce the influence of noise, thereby extracting more stable and reliable features. ReliefF can automatically filter out the features most relevant to hand motion recognition, solve the problem of sEMG signal feature redundancy, and simplify subsequent processing. Combining the optimized features of ReliefF and the fast signal processing capability of VMD, this method supports the implementation of a real-time hand motion recognition system. In sEMG-based hand action recognition, the combination of VMD and ReliefF can provide a more accurate, fast and efficient solution, which will help the development of applications such as intelligent human-computer interaction and rehabilitation training.

The remaining sections of this paper are structured as follows: Section 2 presents the materials and methods. Section 3 presents the results. Section 4 discusses these results in depth. Finally, Section 5 is conclusion.

## 2. Materials and methods

### Dataset

**Fig 1:**
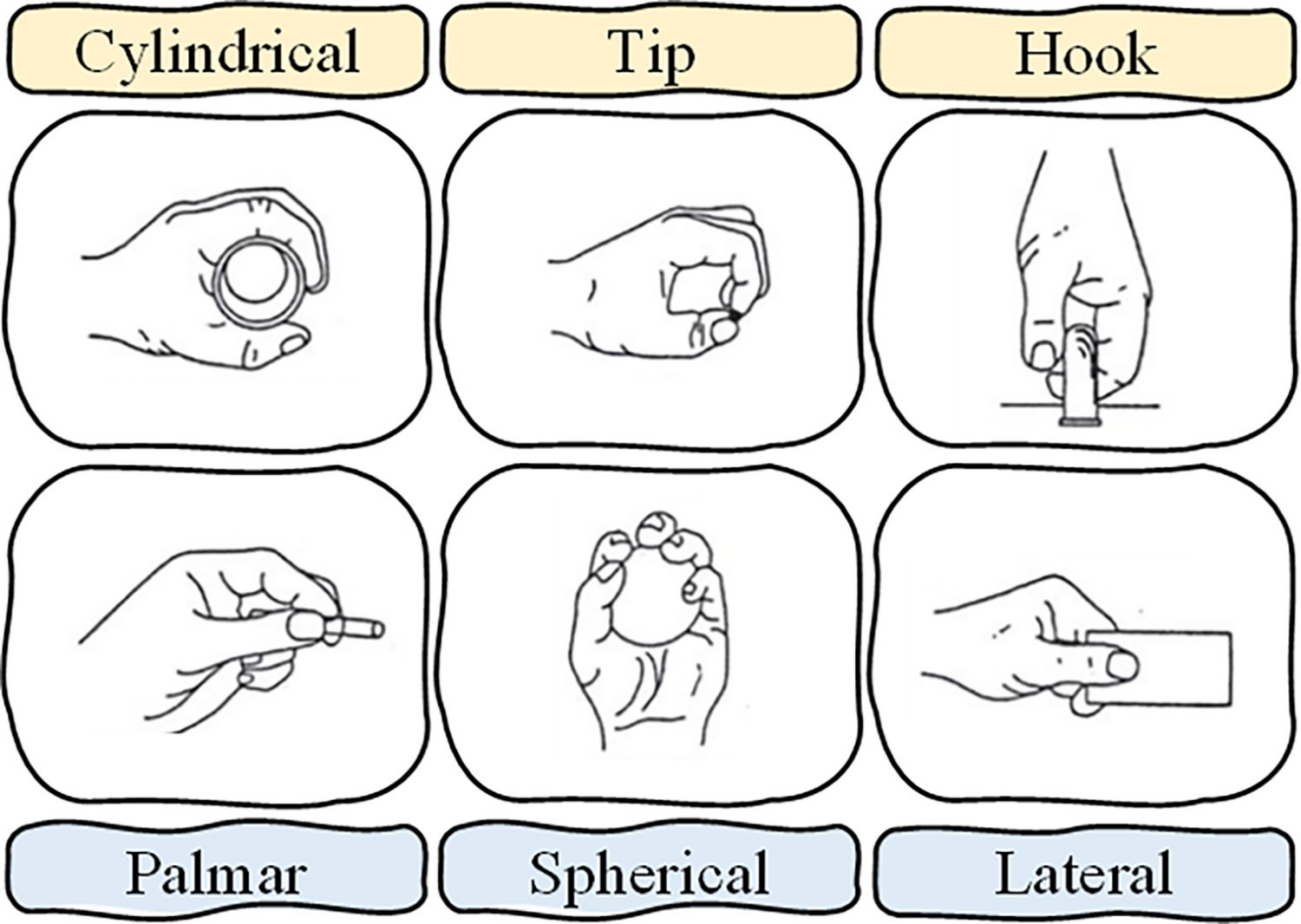
Basic hand movements.

The dataset considered in this work was obtained from the UCI machine learning repository, titled sEMG for Basic Hand movements Data Set. The dataset can be accessed from the following URL: https://archive.ics.uci.edu/ml/datasets/sEMG+for+Basic+Hand+movements#. It was collected at a sampling rate of 500 Hz. To ensure data quality, the signals underwent preprocessing steps. A Butterworth Band Pass filter with cutoff frequencies at 15 Hz and 500 Hz was applied to remove unwanted frequencies, and a notch filter at 50 Hz was used to eliminate line interference artifacts. The data were captured using two Differential EMG Sensors and transmitted to a 2-channel EMG system by Delsys Bagnoli Handheld EMG Systems. The experiment involved participants performing repeated grasps of various items, including cylindrical (CY), tip (TI), hook (HO), palmar (PA), spherical (SP), and lateral (LA) objects, as depicted in Fig 1. The subjects had control over the speed and force exerted during the grasping motions. The forearm sEMG electrodes (Flexor Capri Ulnaris and Extensor Capri Radialis) were secured with elastic bands, along with a reference electrode in the middle, to capture muscle activation information.

Two different databases are included:

1. 5 healthy subjects (two males and three females) of the same age approximately (20 to 22-year-old) conducted the six grasps for 30 times each. The measured time is 6 sec.
2. 1 healthy subject (male, 22-year-old) conducted the six grasps for 100 times each for 3 consecutive days. The measured time is 5 sec.

**Fig 2:**
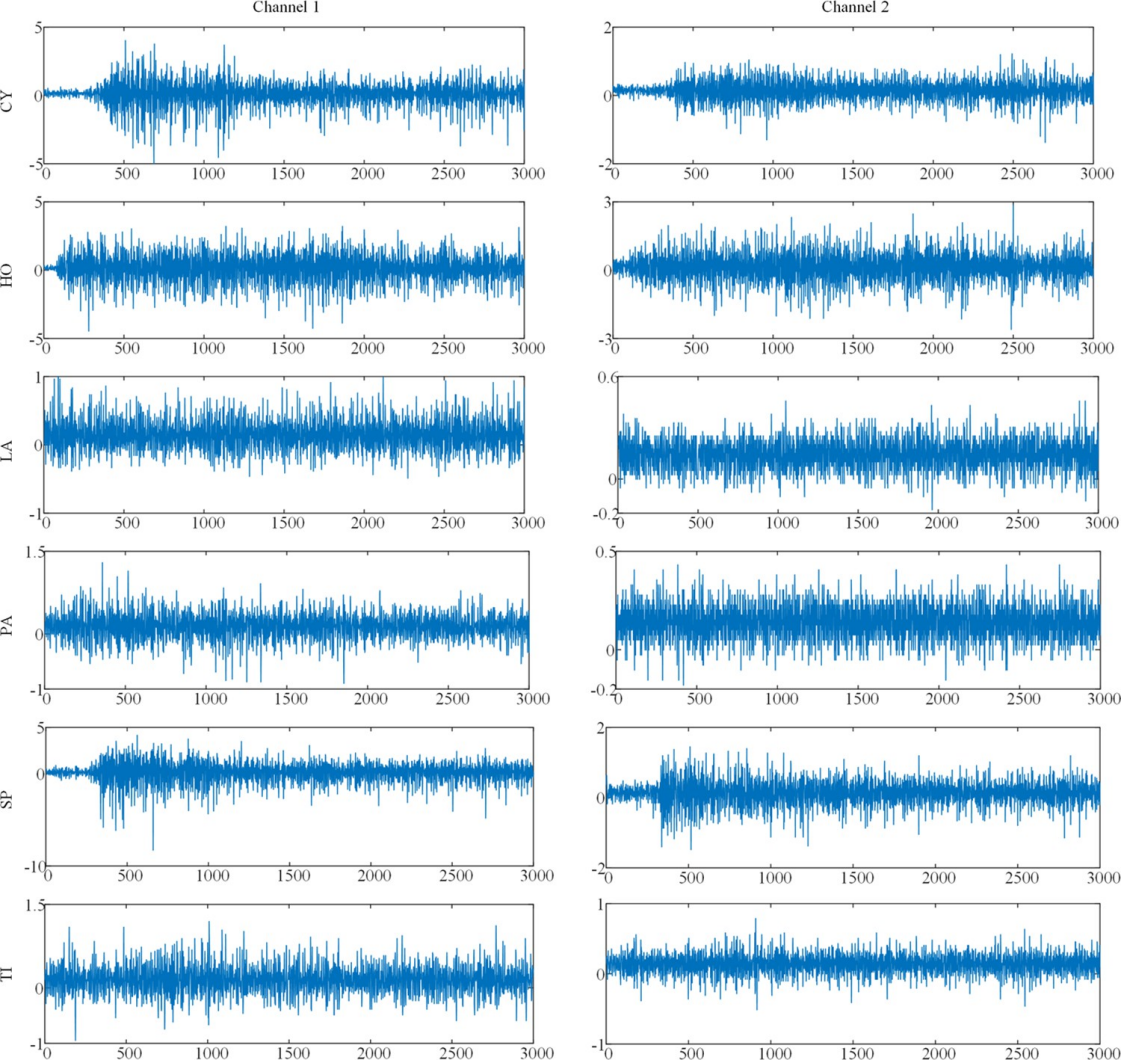
The sample of sEMG signals recorded for each hand movement from female_1.

The dataset used in this work is dataset 1. More details of the data set are introduced in the research [62]. The sEMG samples of different hand movements analyzed in this work are shown in Fig 2. In addition, a sliding window with a window size of 2000 and an increment of 80 was used to process the dataset to extract samples for subsequent decomposition and feature extraction operations.

### Methods

**Fig 3:**
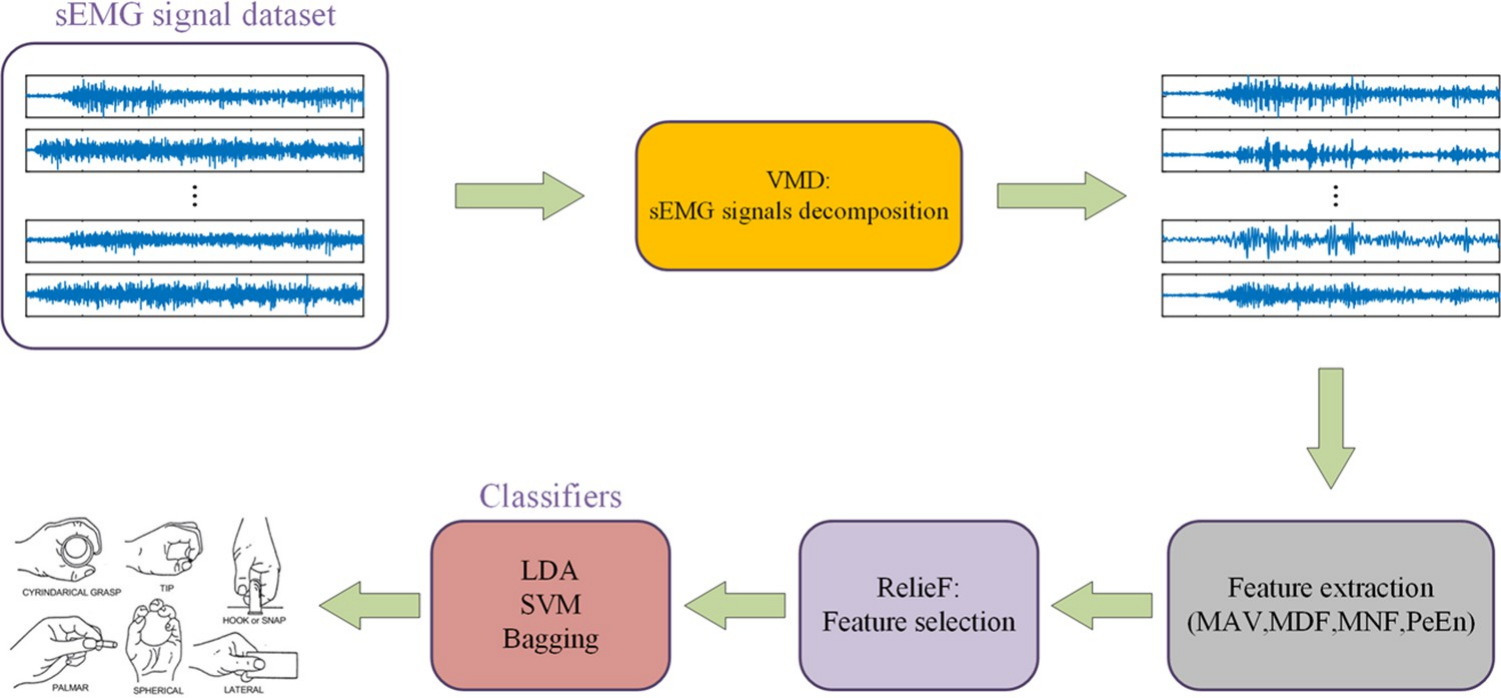
The framework of the method proposed in this work.

The proposed approach consists of several key components, namely sEMG signal acquisition, signal decomposition, feature extraction, feature selection, and hand movement recognition. The complete workflow of the proposed method is illustrated in Fig 3.

#### Variational mode decomposition

The VMD is a method for signal decomposition and feature extraction. VMD utilizes variational principles to decompose an input signal into multiple modes and estimate the amplitude and frequency of each mode [63]. Compared with traditional mode decomposition methods, VMD has better adaptability and robustness. Through VMD, a signal can be represented as a superposition of multiple modes, each mode corresponding to different frequency and amplitude characteristics. This decomposition method can extract local features in the signal, and has a strong ability to suppress noise and interference. VMD works by iteratively optimizing an energy function based on the local frequency and amplitude of the signal. Through an iterative process, VMD gradually extracts the individual modes of the signal and estimates the frequency and amplitude of each mode. Ultimately, the original signal can be reconstructed by superimposing the individual modes. Through the decomposition results of VMD, the modal components in different frequency ranges can be obtained, these components can provide local characteristic information about the signal. These decomposed modal components can be used for feature extraction, signal analysis, pattern recognition and other tasks, thereby improving the performance of signal processing and data analysis.

#### Feature extraction

The extraction of features from sEMG signals has a direct impact on the performance of hand motion recognition [64]. The TD feature intuitively reflects the instantaneous changes of muscle contraction, the FD feature shows the frequency distribution of muscle activity, and the entropy feature quantifies the complexity or uncertainty of the sEMG signal. Since the sEMG signals generated by different hand movements show different feature distributions in the TD and FD, combining the TD and FD features can more comprehensively describe the characteristics of the sEMG signal, thereby improving the accuracy of hand movements recognition. The entropy feature serves as supplementary information to help distinguish hand movements s that are difficult to distinguish in the TD and FD features. The fusion of multiple features can further enhance the robustness and recognition performance of the model. Therefore, four feature extraction methods were selected in this work: one from the TD group (MAV), two from the FD group (MDF and MNF), and one from the entropy group (PeEn) [65,66]. The mathematical expressions as follows:

Mathematically, MAV can be expressed as:

$$\mathrm{MAV}=\frac{1}{N}\sum_{i=1}^{N}\left|x_{i}\right|$$ (1)

Where *N* is the length of the signal and *i* is the *i*th sample point.

Mathematically, MDF can be expressed as:

$$\sum_{j=1}^{MDF}P_{j}=\sum_{j=MDF}^{M}P_{j}=\frac{1}{2}\sum_{j=1}^{M}P_{j}$$ (2)

Where *f*_*j*_ is the frequency of the spectrum, *P*_*j*_ is the signal power spectrum, and *M* is the length of the frequency bin.

Mathematically, MNF can be expressed as:

$$\mathrm{MNF}=\sum_{j=1}^{M}f_{j}P_{j}/\sum_{j=1}^{M}P_{j}$$ (3)

The phase space of the time series was reconstructed, reconstruction component was rearranged in the reconstruction matrix in ascending order, the permutations and combinations of the new sequence is one of m!. The probability of occurrence of each sequence was calculated as *P*_1_, *P*_2_, *…*, *P*_k_.

Mathematically, PeEn can be expressed as:

$$H_{p}(m)=-\sum_{j=1}^{m!}P_{j}\ln P_{j}$$ (4)

#### Feature selection

The ReliefF algorithm is widely used in machine learning [67,68]. The ReliefF algorithm has no restrictions on data types and is a feature weighting algorithm that gives higher weights to all features that are highly correlated with the category. The algorithm operates by randomly selecting a sample R, if the distance between R and the nearest neighbor sample set on a particular feature is smaller than the distance between R and the farthest neighbor sample set, the feature is considered valuable for distinguishing between classes, and its weight is increased. Conversely, if the distance is larger, the weight of the feature is reduced. Repeat this process m times to calculate the average weight of each feature. A higher weight indicates a stronger classification ability, while a lower weight indicates a weaker classification ability for the corresponding feature. When evaluating feature importance, the ReliefF algorithm can effectively ignore noise features that have little impact on classification results, thereby enhancing the robustness of the model. Unlike linear dimensionality reduction methods such as principal component analysis, ReliefF better preserves key information in the original data by retaining features that contribute significantly to classification rather than reducing the dimension through linear transformation. In addition, compared with traditional feature selection algorithms, the ReliefF algorithm is able to handle multi-category classification problems, which is particularly applicable to the six hand movements recognition methods in this work.

#### Classification

After the feature extraction process, a feature set is constructed using the extracted features from the sEMG signal. This feature set is then used for hand movement recognition with the help of three machine learning algorithms. These algorithms are briefly described below:

The basic principle of LDA classifier is to project samples onto a straight line so that the projection points of similar samples are as close as possible and the projection points of heterogeneous samples are as far as possible [56,69]. The LDA classifier offers several advantages, including robustness, ease of training, and low computational cost [70]. In this work, the quadratic kernel function is selected for the LDA classifier to improve hand motion recognition performance.

The SVM classifier aims to find a hyperplane that separates the data into different classes by transforming it into a higher-dimensional space [71]. SVM is known for its efficiency and reliability, particularly in limited data scenarios [54]. In this work, the SVM classifier is employed with a quadratic polynomial kernel function for improved hand motion recognition performance.

Bagging classifier aims to reduce generalization error by combining multiple models [72]. The approach involves training individual models independently and aggregating their predictions through voting [73]. In this work, the decision tree is chosen as the base classifier.

#### Performance evaluation

Ten-fold cross validation was used to evaluate the proposed method, and the evaluation indicators were accuracy, precision, recall and F1-score [74]. The evaluation process and the calculation of the four evaluation indicators are based on a single subject’s data. Readers are referred to [75] for further details.

## 3. Results

The proposed approach consists of several key components, namely sEMG signal acquisition, signal decomposition, feature extraction, feature selection, and hand movement recognition. The complete workflow of the proposed method is illustrated in Fig 3.

### Decomposition performance of VMD

**Fig 4:**
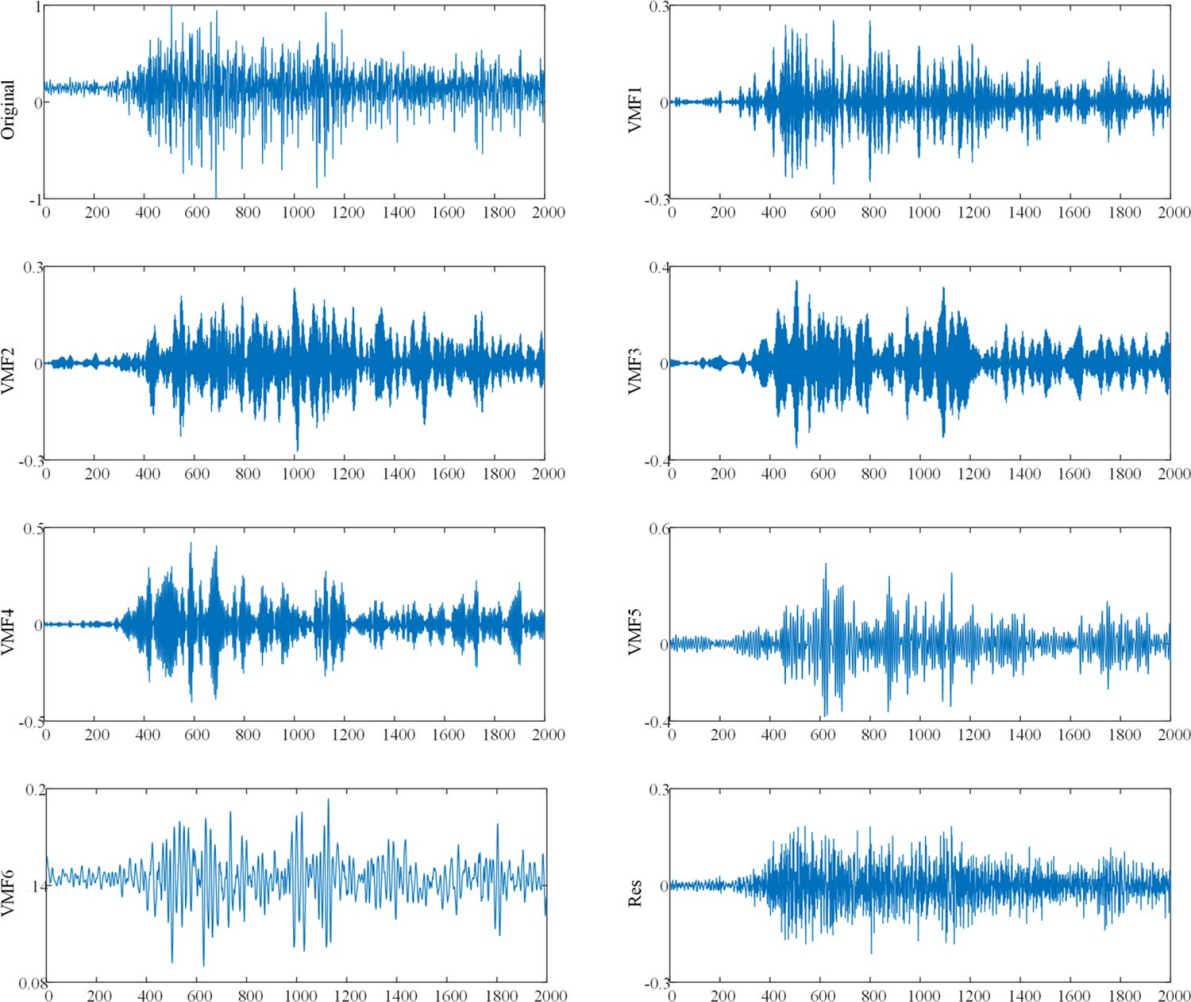
Example of sEMG signal decomposed by VMD. Original is the original sEMG signal. VMF1-VMF6 and Res are the 6 sub-band components and residual signals.

In this work, the VMF decomposition method is used to decompose the sEMG signal, and futher accurately extract the features that characterize hand movements. Fig 4 shows an example of six sub-band components and one residual component obtained by decomposing the sEMG signal by the VMD method, where the number of sub-band components decomposed by the VMD decomposition method is set to six. Fig 4 shows that the sub-band components obtained by decomposing the sEMG signal by the VMD decomposition method have unique properties compared with the original sEMG signal. These sub-band components are more conducive to extracting the features required for hand motion classification.

### The number of VMFs selection

As can be seen from Tables 1 and 2, for the LDA classifier and the SVM classifier, when the number of VMFs is around 10, all subjects achieved the best average recognition accuracy of hand movements. For the LDA classifier, subject 1, subject 4 and subject 5 achieved the highest recognition accuracy when the number of VMFs was 10, which were 98.84%, 99.01% and 99.24%, respectively, and subject 2 and subject 3 have the highest recognition accuracy when the number of VMFs was 11, which were 98.77% and 98.45%, respectively. For the SVM classifier, subject 1 have the highest recognition accuracy of 99.39% when the number of VMFs was 9, subject 2 have the highest recognition accuracy of 99.08% when the number of VMFs was 11, and subject 3, subject 4 and subject 5 achieved the highest recognition accuracy when the number of VMFs was 10, which were 98.66%, 99.31% and 99.68%, respectively. Table 3 shows that for the Bagging classifier, subject 1 and subject 2 achieved the highest recognition accuracy when the number of VMFs was 10, which were 96.79% and 96.76%, respectively, subject 3, subject 4 and subject 5 achieved the highest recognition accuracy when the number of VMFs was 5, which were 97.70%, 98.21% and 99.29%, respectively. For all subjects, the average recognition accuracy of the three classifiers for hand movements decreases gradually after reaching the highest recognition accuracy with the increase of the number of VMFs. Comprehensively considering the recognition accuracy of the three classifiers based on the number of different VMFs, when the number of VMFs is 10, the hand movement recognition performance of most subjects is the best. In addition, the comprehensive classification performance of the three classifiers is SVM classifier, LDA classifier and Bagging classification in descending order. Therefore, our next work is carried out based on the number of VMFs is 10 and the SVM classifier.

**Table 1:** The average accuracy of the different numbers of VMFs by the LDA classifier.

Subject	4	5	6	7	8	9	10	11	12	13	14	15	16
**Subject 1**	87.56	93.67	96.48	97.06	98.27	98.56	98.84	98.56	98.03	98.07	98.06	97.68	97.58
**Subject 2**	85.73	92.42	96.23	96.62	97.32	98.62	98.76	98.77	98.74	98.36	98.15	97.89	97.47
**Subject 3**	95.96	95.94	97.25	97.56	97.31	98.15	98.36	98.45	98.09	98.08	97.12	96.88	96.86
**Subject 4**	92.63	96.88	98.31	98.66	98.72	98.73	99.01	98.79	98.42	98.40	98.36	98.07	97.86
**Subject 5**	98.89	99.11	99.19	99.23	99.23	99.24	99.24	99.02	98.95	98.88	98.51	98.47	98.41

**Table 2:** The average accuracy of the different numbers of VMFs by the SVM classifier.

Subject	4	5	6	7	8	9	10	11	12	13	14	15	16
**Subject 1**	95.62	97.18	97.86	98.17	99.08	99.39	99.37	99.14	99.12	99.11	99.08	99.06	99.02
**Subject 2**	92.08	95.3	96.52	97.64	97.89	98.73	98.94	99.08	98.74	98.72	98.71	98.69	98.56
**Subject 3**	97.78	98.35	98.43	98.46	98.52	98.63	98.66	98.57	98.56	98.55	98.55	98.41	98.35
**Subject 4**	97.96	97.72	98.66	98.67	98.84	99.12	99.31	99.02	98.80	98.74	98.72	98.70	98.65
**Subject 5**	99.06	99.28	99.28	99.36	99.42	99.42	99.68	99.36	99.35	99.35	99.16	99.08	99.02

**Table 3:** The average accuracy of the different numbers of VMFs by the Bagging classifier.

Subject	4	5	6	7	8	9	10	11	12	13	14	15	16
**Subject 1**	93.79	95.73	95.74	96.22	96.24	96.28	96.79	96.74	96.16	95.54	95.04	95.01	94.34
**Subject 2**	92.68	94.79	94.86	94.89	94.91	94.91	96.76	96.23	96.03	94.37	94.23	94.18	94.12
**Subject 3**	97.48	97.70	97.32	97.31	97.25	97.12	96.77	96.41	96.28	96.26	96.12	95.32	95.26
**Subject 4**	98.11	98.21	98.08	98.05	97.22	97.13	97.01	96.13	96.13	96.11	95.62	95.59	94.57
**Subject 5**	98.53	99.29	98.42	98.33	98.32	98.01	97.99	97.87	97.81	97.76	97.36	97.12	96.79

### Feature dimension selection

In order to select a feature space that can effectively represent hand movements, this work uses the ReliefF to perform feature selection on the feature space composed of 88 extracted features. As Table 4 shows that subject 1, subject 2 and subject 5 achieved the highest average recognition accuracy when the feature space dimension was 42, which were 99.54%, 98.83% and 99.73%, respectively, subject 3 and subject 4 had the highest average recognition accuracy when the feature space was 48, which were 98.63% and 99.06%, respectively. Fig 4 shows that the average recognition accuracy of hand movements of all subjects increases with the increase of the feature space dimension, and slightly decreases after reaching the highest recognition accuracy. Comparing the hand motion recognition accuracy of 8 different low-dimensional feature spaces comprehensively, the classification performance of 42-dimensional feature space is the best.

**Table 4:** Average recognition accuracy of different dimensional feature spaces.

Subject	The dimension of the feature space							
	16	24	30	36	42	48	54	60
**Subject 1**	97.02%	98.31%	99.42%	99.49%	99.54%	99.31%	99.28%	99.22%
**Subject 2**	92.91%	97.23%	98.23%	98.62%	98.83%	98.76%	98.69%	98.66%
**Subject 3**	97.09%	98.31%	98.47%	98.52%	98.57%	98.63%	98.41%	98.37%
**Subject 4**	96.33%	97.61%	98.80%	98.95%	99.04%	99.06%	98.97%	98.91%
**Subject 5**	97.58%	99.32%	99.35%	99.38%	99.73%	99.67%	99.61%	99.42%

### Evaluation of sEMG signal recognition performance

Tables 5–9 show the accuracy, precision, recall and F1 for all subjects. The recognition performance of subject 1 and subject 3 is that the accuracy, precision, recall and FI1 of HO are the best, and the accuracy, precision, recall and F1 of LA are the lowest. The recognition performance of subject 2 is that the accuracy, precision, recall and F1 of HO are the best, the accuracy, precision and F1 of LA are the lowest, and the recall of PA is the lowest. The recognition performance of subject 4 is as follows, HO has the best accuracy, precision, recall and F1, PA has the lowest accuracy, recall and F1, and TI has the lowest precision. Compared with other subjects, subject 5 performed the best in comprehensive recognition, TI had the highest accuracy, and SP had the lowest precision and recall. Combining the recognition performance of each hand movement of all the subjects, the proposed method has the best recognition performance for HO and the worst recognition performance for TI.

**Table 5:** The recognition performance of subject 1.

Grasps	Accuracy	Precision	Recall	F1
**CY**	99.83%	99.74%	99.23%	0.99
**HO**	99.96%	99.74%	100%	1.00
**LA**	99.70%	99.48%	98.74%	0.99
**PA**	99.74%	98.73%	99.74%	0.99
**SP**	99.87%	99.49%	99.74%	1.00
**TI**	99.87%	99.74%	99.49%	1.00

**Table 6:** The recognition performance of subject 2.

Grasps	Accuracy	Precision	Recall	F1
**CY**	99.70%	99.23%	98.97%	0.99
**HO**	99.87%	99.74%	99.49%	1.00
**LA**	99.02%	96.69%	97.44%	0.97
**PA**	99.06%	97.18%	97.18%	0.97
**SP**	99.79%	99.23%	99.49%	0.99
**TI**	99.83%	99.74%	99.23%	0.99

**Table 7:** The recognition performance of subject 3.

Grasps	Accuracy	Precision	Recall	F1
**CY**	99.70%	98.98%	99.23%	0.99
**HO**	100%	100%	100%	1.00
**LA**	98.97%	96.92%	96.92%	0.97
**PA**	99.23%	96.97%	98.46%	0.98
**SP**	99.70%	99.23%	98.97%	0.99
**TI**	99.74%	100%	98.46%	0.99

**Table 8:** The recognition performance of subject 4.

Grasps	Accuracy	Precision	Recall	F1
**CY**	99.96%	99.74%	100%	1.00
**HO**	100%	100%	100%	1.00
**LA**	99.66%	98.97%	98.97%	0.99
**PA**	99.32%	98.45%	97.44%	0.98
**SP**	99.96%	100%	99.74%	1.00
**TI**	99.40%	97.72%	98.72%	0.98

**Table 9:** The recognition performance of subject 5.

Grasps	Accuracy	Precision	Recall	F1
**CY**	99.87%	99.24%	100%	1.00
**HO**	99.91%	99.49%	100%	1.00
**LA**	99.87%	100%	99.23%	1.00
**PA**	99.96%	99.74%	100%	1.00
**SP**	99.83%	100%	98.97%	0.99
**TI**	99.96%	99.74%	100%	1.00

## 4. Discussion

Different muscle activity information contained in sEMG signals often overlap, and different numbers of muscle activity subcomponents will result in different hand movements recognition performances. Evaluating the hand movements recognition performance of different numbers of VMFs and finding out the independent subcomponents produced by specific muscles or muscle fiber groups are of great significance to improving the performance of hand movements recognition. The sEMG signal is decomposed into multiple sub-band signals by the VMD decomposition method to further extract the essential components that characterize hand movements. Since the number of VMFs can be set artificially, it has great flexibility. Therefore, this work evaluates the performance of hand motion recognition based on different numbers of VMFs (from VMFs = 4 to VMFs = 16). As shown in Tables 1–3, when the number of VMFs is small (such as VMFs = 4), the recognition performance of hand movements is poor. This is interpreted as the number of VMFs set is small, and the VMD decomposition method cannot effectively extract the effective components representing hand movements in the sEMG signal. As the number of decomposed VMFs increases, the hand motion recognition performance of the proposed method improves. When the number of VMFs reached an appropriate number, the method achieves the best recognition performance. Next, when the number of VMFs continues to increase, the hand action recognition accuracy decreases slightly. This may be explained as follows: The sEMG signal is produced by the combined action of superficial muscle EMG and electrical activity of the nerve trunks on the surface of the skin. In addition, there are noises caused by factors such as muscle fatigue and electrode displacement in the process of sEMG signal acquisition. When the number of VMFs increases, it is inevitable to introduce noise components that have nothing to do with hand movements. However, when the number of decomposed VMFs is larger, the hand motion recognition accuracy may have better recognition performance, but the computational overhead increases. Comprehensively considering the recognition performance and calculation cost, this work sets the number of VMFs obtained by decomposing sEMG signals to 10.

The feature space of this work is constituted by extracting MAV, MDF, MNF and PeEn from the sub-band components. In order to reduce the dimensionality of the feature space while ensuring the performance of hand motion recognition, ReliefF is used to select the features that best represent the hand motion intention. As shown in Table 4, 8 low-dimensional feature spaces are evaluated for hand motion recognition performance. When the dimensionality is low (such as 16, 24, etc.), the recognition accuracy for hand movements is low, which may be due to the fact that the low-dimensional feature space cannot effectively reflect the intention of hand movements. When the dimension increases, the hand motion information contained in the feature space also increases, and the proposed method also improves the accuracy of hand motion recognition. When the dimension of the feature space is further increased (such as 54, 60, etc.), the proposed method is not ideal for improving the recognition accuracy, which can be explained by the redundancy between the features of the high-dimensional feature space lead to a decrease in recognition accuracy. Based on the analysis of hand movement recognition accuracy of all subjects shown in Table 4, this work selects a 42-dimensional low-dimensional feature space and feeds it into the classifier to recognize hand movements. In addition, we performed the statistical analysis of the Kruskal-Wallis test on the 42-dimensional low-dimensional feature space [72]. The results of statistical analysis show that the features in the 42-dimensional low-dimensional feature space have statistical differences (p<0.05), which further shows that the selected 42-dimensional low-dimensional feature space retains most of the information about hand movement intentions while reducing the redundancy between features.

As shown in Table 10, the recognition performance of the method proposed is compared with several existing studies. For the studies of Subasi, Alharbi (21), Iqbal, Fattah (23) and Sapsanis, Georgoulas (50), the average recognition accuracy of each subject is lower than that of the method proposed. In the study by Akben (62), the recognition accuracy of subject 4 is better than that of the method proposed, but the recognition accuracy of the rest of the subjects is lower than that of the method proposed. Table 10 shows that the average hand motion recognition accuracy of all existing studies is lower than the average hand motion recognition accuracy of the method proposed. Compared with the existing research, the average recognition accuracy of the method proposed reaches 99.14%, which proves the superiority of proposed method in the field of hand motion recognition.

**Table 10:** Comparison of the proposed method with several existing studies.

Research	Features extraction	Classifier	Subject 1	Subject 2	Subject 3	Subject 4	Subject 5	Average
Subasi, Alharbi (21)	WPD	Rotation forest	95.56%	88.88%	92.22%	92.22%	98.33%	93.44%
Iqbal, Fattah (23)	SVD and PCA	KNN	82.78%	87.67%	83.11%	90.00%	90.00%	86.71%
Sapsanis, Georgoulas (50)	EMD	Linear classifier	87.25%	88.05%	85.83%	90.42%	94.80%	89.21%
Akben (62)	Histogram approach	k-means	93.04%	86.66%	97.00%	99.23%	97.66%	94.72%
Proposed method	VMD and ReliefF	SVM	99.54%	98.83%	98.57%	99.04%	99.73%	99.14%

**Fig 5:**
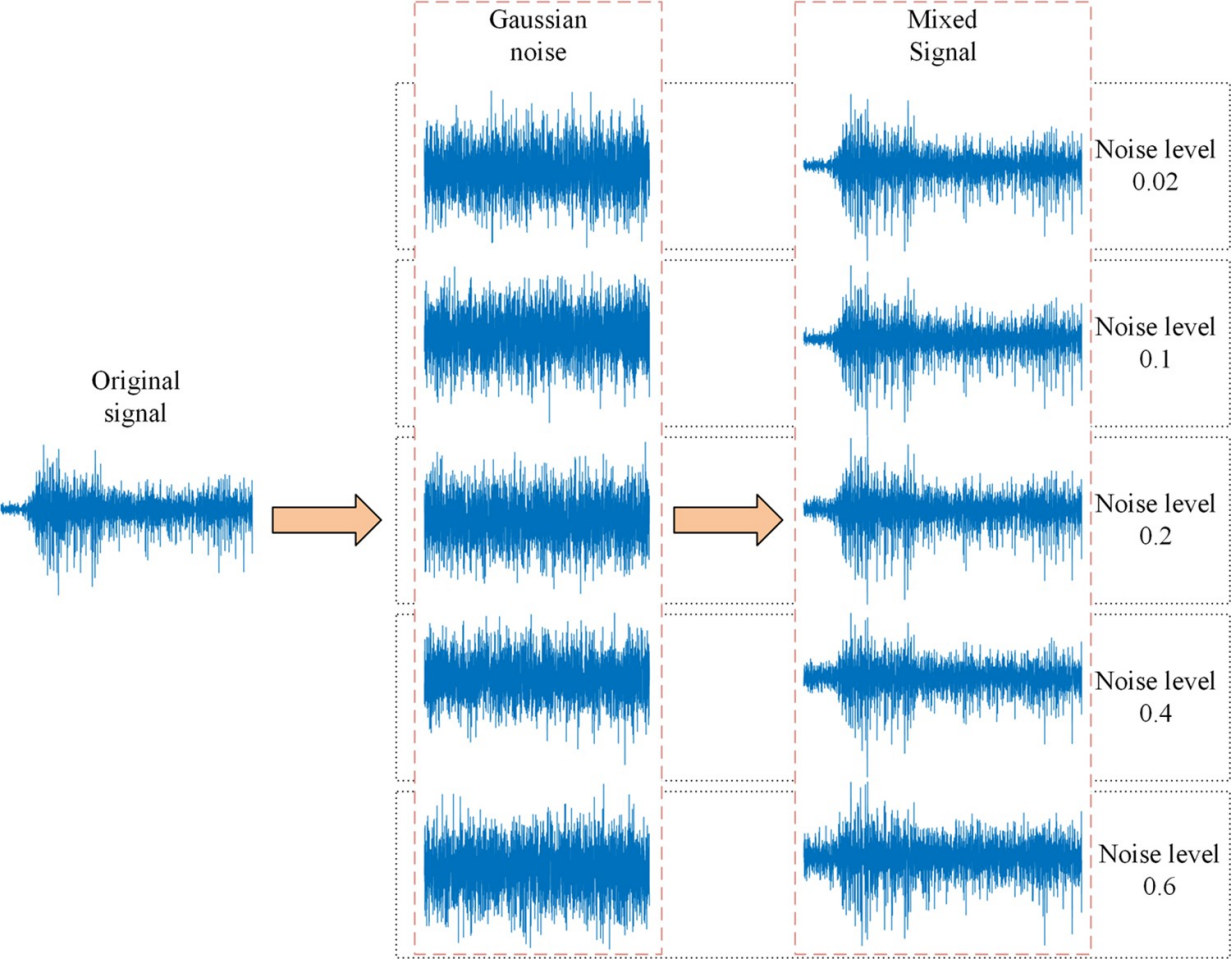
The example of the mixing process of the original sEMG signal and Gaussian white noise.

**Fig 6:**
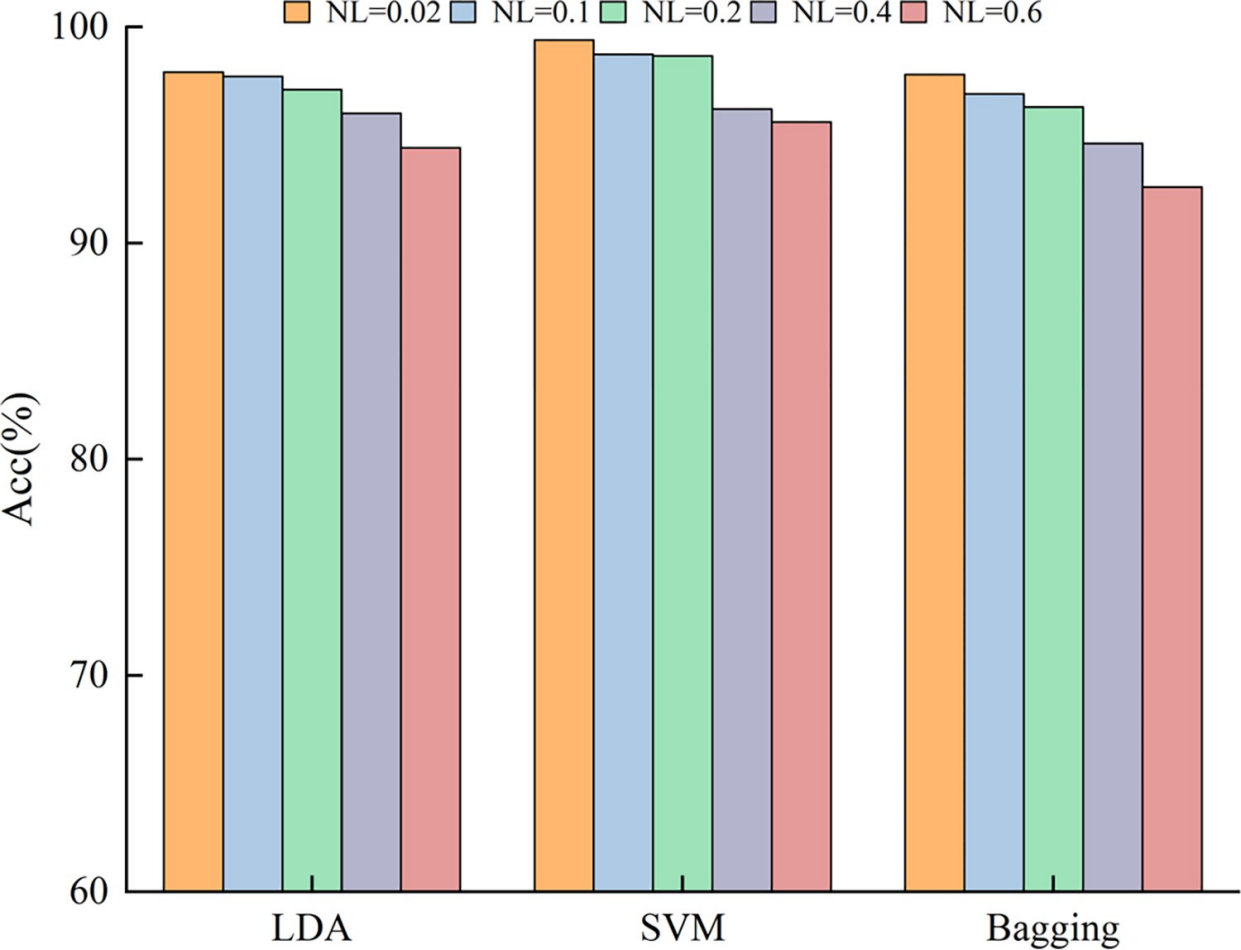
The hand movement recognition accuracy under different noise levels. NL = 0.02 means the Gaussian white noise level is 0.02, and so on.

Due to factors such as force level changes and electrode shift, the collected sEMG signals will be contaminated by noise, which in turn affects the performance of the proposed method for hand movement recognition. Based on this, this work takes the data of Subject 1 as an example, adds different levels of Gaussian white noise to the original sEMG signal, and inputs the perturbed sEMG signal into the proposed method for hand movement recognition to further evaluate the robustness of the proposed method. Five different levels of Gaussian white noise are set in this work, which are 0.02, 0.1, 0.2, 0.4 and 0.6 respectively. Fig 5 shows an example of the mixing process of the original sEMG signal and Gaussian white noise. Fig 6 shows the hand movement recognition accuracy under different noise levels. As can be seen from Fig 6, the SVM classifier has the best robustness, followed by the LDA classifier and the Bagging classifier. Even when the Gaussian white noise level reaches 0.6, the SVM classifier can still achieve a hand movement recognition accuracy of more than 95%. In summary, the proposed method still shows good generalization ability in the face of inevitable data perturbations such as electrode displacement, sensor noise and muscle fatigue, and has the potential for practical application.

Although the public dataset used in this work shows the characteristics of class balance, in actual applications, factors such as environmental changes and individual differences may lead to class imbalance in surface electromyography data. This imbalance phenomenon may cause the model to tend to learn a larger number of categories, thereby ignoring a smaller number of categories, and ultimately leading to a decrease in the recognition accuracy of minority classes and impaired overall performance in actual tests. In order to solve this problem, a variety of methods have been proposed to deal with class imbalance, including data enhancement, resampling technology, and ensemble learning. Data enhancement methods effectively increase the number of minority class samples by transforming minority class samples, such as scaling and adding noise, thereby achieving a balanced dataset. The oversampling method in the resampling technology can increase the number of minority class samples by copying existing samples or generating new samples. Correspondingly, the undersampling method achieves data balance by reducing the number of majority class samples, but this method may lead to the loss of important information in applications. The ensemble learning method combines the prediction results of multiple models to reduce the bias of a single model and improve the recognition ability of minority classes. In practical applications, it is necessary to combine specific application scenarios and select appropriate methods to solve the class imbalance problem, so as to improve the overall performance of the hand motion recognition system and enhance the user’s interactive experience, especially in the fields of assistive technology and human-computer interaction.

The hand movements recognition based on sEMG signals proposed in this work will show great potential in the field of healthcare. This method captures the electrical signals of muscle activity and converts them into specific hand movement information, providing an innovative rehabilitation training method for patients with motor dysfunction. For patients with impaired motor function due to stroke or nervous system damage, the hand rehabilitation equipment based on the method proposed in this work can accurately guide rehabilitation training and gradually restore hand motor function. In addition, the proposed method will also show significant advantages in prosthetic control. Based on the recognition of the sEMG signals of the patient’s residual limb muscles, the prosthesis can achieve natural hand movements, thereby significantly improving the patient’s quality of life. At the same time, the hand movements recognition based on sEMG signals proposed in this work can also assist doctors in diagnosing and evaluating muscle function, providing a scientific basis for formulating personalized treatment plans.

## 5. Conclusion

In this work, we propose a novel hand motion recognition method based on the combination of VMD and ReliefF. The VMF hand motion recognition performance under different numbers of VMFs is evaluated. The evaluation results show that when the number of VMFs is 10, the proposed method has better performance in hand motion recognition. In addition, the ReliefF algorithm is used to perform feature selection on the extracted feature space, and a 42-dimensional low-dimensional feature space that effectively represents hand movements is selected. The SVM classifier is used to recognize 6 hand movements and achieves an average recognition accuracy of 99.14%. Compared with existing researches, the proposed method has obvious advantages in the accuracy of hand motion recognition, indicating that it has application potential in healthcare fields such as motion intention detection and intelligent prosthetic control.

This suggests that our method can significantly contribute to rehabilitation medicine by improving the effectiveness of prosthetic devices and enhancing the quality of life for individuals with hand disabilities. The ability to accurately interpret hand motion intentions can facilitate more natural and intuitive control of prosthetic hands, thereby aiding in the physical and psychological rehabilitation of patients.

## Supporting information

S1 File (ZIP)
