# Supplementary material for: Application of a sEMG hand motion recognition method based on variational mode decomposition and ReliefF algorithm in rehabilitation medicine
Source: PLoS One. 2024 Nov 27;19(11):e0314611. doi: 10.1371/journal.pone.0314611 (PMC11602058; doi:10.1371/journal.pone.0314611)
Supplement: S1 File — (ZIP) [file pone.0314611.s001.zip › Supporting Information/Data Description.docx]

The dataset considered in this work was obtained from the UCI machine learning repository, titled sEMG for Basic Hand movements Data Set. The dataset can be accessed from the following URL: https://archive.ics.uci.edu/ml/datasets/sEMG+for+Basic+Hand+movements#.

The dataset used in this work is already included in this folder.
